# Expression Profiling along the Murine Intestine: Different Mucosal Protection Systems and Alterations in *Tff1*-Deficient Animals

**DOI:** 10.3390/ijms241612684

**Published:** 2023-08-11

**Authors:** Franz Salm, Eva B. Znalesniak, Aikaterini Laskou, Sönke Harder, Hartmut Schlüter, Werner Hoffmann

**Affiliations:** 1 Institute of Molecular Biology and Medicinal Chemistry, Otto-von-Guericke University Magdeburg, Leipziger Str. 44, 39120 Magdeburg, Germany; 2 Section Mass Spectrometry and Proteomics, Diagnostic Center, University Medical Center Hamburg-Eppendorf, Martinistr. 52, 20246 Hamburg, Germany

**Keywords:** trefoil factor, TFF, Fcgbp, colon cancer, mucin, Muc6, goblet cell, Brunner gland, innate immunity, reactive oxygen species

## Abstract

Tff1 is a typical gastric peptide secreted together with the mucin, Muc5ac. *Tff1*-deficient (*Tff1*^KO^) mice are well known for their prominent gastric phenotype and represent a recognized model for antral tumorigenesis. Notably, intestinal abnormalities have also been reported in the past in these animals. Here, we have compared the expression of selected genes in *Tff1*^KO^ mice and their corresponding wild-type littermates (RT-PCR analyses), focusing on different mucosal protection systems along the murine intestine. As hallmarks, genes were identified with maximum expression in the proximal colon and/or the duodenum: *Agr2*, *Muc6*/*A4gnt*/*Tff2*, *Tff1*, *Fut2*, *Gkn2*, *Gkn3*, *Duox2*/*Lpo*, *Nox1*. This is indicative of different protection systems such as Tff2/Muc6, Tff1-Fcgbp, gastrokines, fucosylation, and reactive oxygen species (ROS) in the proximal colon and/or duodenum. Few significant transcriptional changes were observed in the intestine of *Tff1*^KO^ mice when compared with wild-type littermates, *Clca1* (*Gob5*), *Gkn1*, *Gkn2*, *Nox1*, *Tff2*. We also analyzed the expression of *Tff1*, *Tff2*, and *Tff3* in the pancreas, liver, and lung of *Tff1*^KO^ and wild-type animals, indicating a cross-regulation of *Tff* gene expression. Furthermore, on the protein level, heteromeric Tff1-Fcgbp and various monomeric Tff1 forms were identified in the duodenum and a high-molecular-mass Tff2/Muc6 complex was identified in the proximal colon (FPLC, proteomics).

## 1. Introduction

The intestinal tract consists of two major segments, i.e., the small intestine (duodenum, jejunum, and ileum) and the large intestine (caecum, colon, and rectum), which differ in their morphology [1]. The lumen is lined by a delicate mucous epithelium, which is protected by different mechanisms, one being the continuous self-renewal from stem and precursor cells [2]. The various parts of the intestine have different physiological functions concerning the digestion of food, the absorption of nutrients, and the excretion of fecal pellets. The number of bacteria drastically increases towards the colon [3,4]. For example, the distal ileum contains about 10^8^ bacteria per milliliter of luminal content and the colon contains about 10^11^ [5]. The mucosa-associated microbiota differ along the intestinal tract, including at least three different bacterial ecosystems with significant differences between the distal ileum and caecum, and also between the ascending colon and the transverse colon [6]. The large number of bacteria in the colon is likely the reason why it is protected by a two-layered mucus barrier, the inner layer not being penetrable for bacteria [5,7]. In contrast, the small intestine is covered by a single mucus layer [5]. The predominant mucin in the murine intestine is Muc2, which is a typical secretory product of different types of goblet cells [8]. Mucin glycosylation is an important element in the regulation of the intestinal microbiota. In mice, the small intestine is dominated by sialylated glycans, whereas in the colon, fucosylation dominates [9]. Other typical secretory products of intestinal goblet cells are the trefoil factor family (TFF) peptide, Tff3; IgG Fc binding protein (Fcgbp); and the calcium-activated chloride channel regulator 1 and metalloprotease Clca1 (previously: Gob5) [10,11,12,13]. Remarkably, at least in humans, TFF3 and FCGBP form disulfide-linked heteromers [10] and there are multiple indications that FCGBP and TFF3-FCGBP play a key role in the innate immune defense of mucous epithelia [12,14,15]. Furthermore, the protein disulfide isomerase, Agr2, is essential for the production of mucus [16]. It is located in the endoplasmic reticulum and also occurs in a secreted form [17].

Another source of intestinal mucous protection are the Brunner glands, which are localized in the proximal duodenum only, and are usually not found beyond the entrance of the pancreatic duct [18]. As a hallmark, they secrete the mucin Muc6, which contains the unusual terminal carbohydrate moiety GlcNAcα1→4Galβ1→R (review: [19]). The key enzyme for the synthesis of the αGlcNAc residue is α1,4-*N*-acetylglucosaminyltransferase encoded by the *A4gnt* gene [19]. The αGlcNAc residue is recognized by the lectin GSA-II from *Griffonia simplicifolia* [20]. Of particular note, the TFF peptide Tff2 is a lectin, which binds to GlcNAcα1→4Galβ1→R and physically stabilizes the mucus barrier by crosslinking Muc6 (review: [21]). The combined expression of the Muc6/A4gnt/Tff2 system is not restricted to Brunner glands, but is also observed in gastric mucous neck and antral gland cells (MNCs, AGCs), and is conserved from frog to human [19,20,21,22]. This explains why Tff2 and Muc6 are co-localized in the gastric mucus [23].

Protection of the intestinal mucosa is also greatly facilitated by extracellular reactive oxygen species (ROS), in particular hydrogen peroxide (H_2_O_2_) and the superoxide anion radical, O_2_**^▪^** ^−^, which are part of the innate immune defense directly attacking microorganisms [24,25]. Furthermore, ROS also trigger signaling cascades important for mucosal healing and regeneration [25]. Generation of these “primary ROS” occurs via the NOX/DUOX family of transmembrane NADPH oxidases in epithelial cells [25,26,27,28]. In the intestine, Nox1 generates superoxide, whereas Duox2 is responsible for the production of extracellular H_2_O_2_ [25]. The latter is than used by secretory lactoperoxidase (Lpo), primarily to oxidize thiocyanate (SCN^−^) into the potent microbicidal component hypothiocyanite (OSCN^−^), which is effective against a wide range of microorganisms (DUOX/H_2_O_2_/LPO/SCN^−^ system) [28]. Excess of extracellular superoxide is destroyed by the extracellular superoxide dismutase Sod3, generating H_2_O_2_ [29]. Further protection systems include gastrokines (Gkn1-3) [30], antimicrobial peptides from Paneth cells of the small intestine [31], and the intestinal immune system [32].

TFF peptides are evolutionary old lectins with important roles in mucosal protection and repair (reviews: [33,34,35]). They even occur in the skin and gastrointestinal (GI) tract of the frog *Xenopus laevis* [36,37]. The most prominent phenotype in mice has been observed after inactivation of the *Tff1* gene (*Tff1*^KO^ mice) [38]. These animals obligatorily develop antral/pyloric adenoma and carcinomas have been detected in about 30% [38]. Thus, Tff1 is considered as an antral tumor suppressor (reviews: [39,40]). Expression profiling of the stomach revealed significant differences in *Tff1*^KO^ mice when compared with the corresponding wild-type animals [41]. Remarkably, also intestinal abnormalities have been reported for *Tff1*^KO^ mice, i.e., enlarged villi and abnormal infiltration of lymphoid cells [38,39]. Thus, we expanded our previous studies [41] to the intestine. Notably, *Tff1* expression has not been detected in the adult murine intestine in the past [42]. However, delivery of Tff1 via engineered *Lactococcus lactis* or via the transgenic expression of TFF1 was found to increase resistance to intestinal damage in mice [43,44]. Here, we present the systematic expression profiling of six different regions of the murine intestine and compare *Tff1*^KO^ mice with the corresponding wild-type littermates at the age of six weeks. The focus is on genes involved in various mucosal protection systems. Furthermore, we present protein data concerning Tff1 in the duodenum and Tff2 in the caecum/colon.

## 2. Results

### 2.1. Expression Profiling of the Murine Intestine (RT-PCR Analysis)

**Figure 1:**
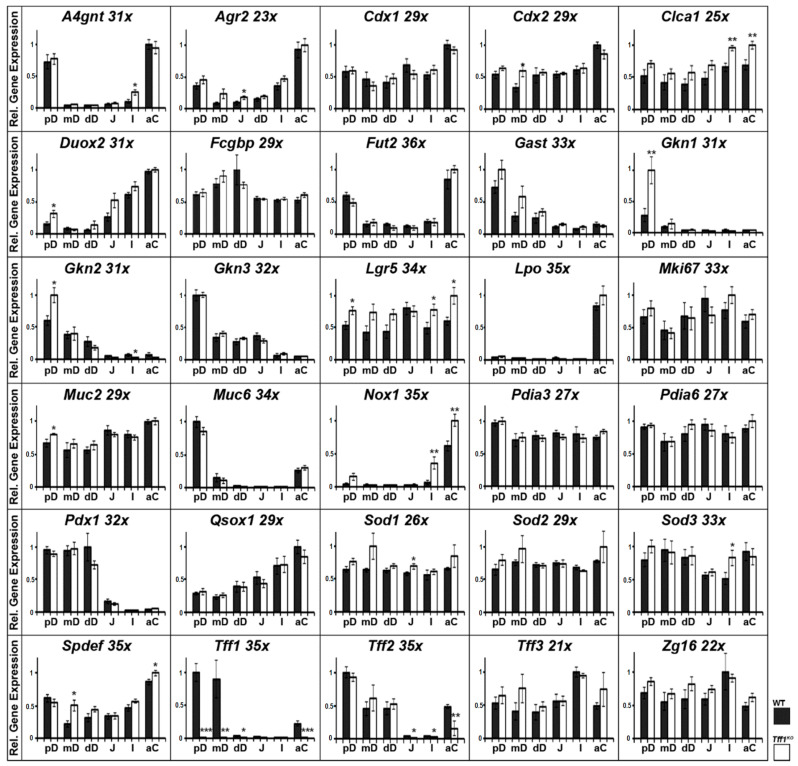
Semi-quantitative RT-PCR analyses. *A4gnt*, *Agr2*, *Cdx1*, *Cdx2*, *Clca1*, *Duox2*, *Fcgbp*, *Fut2*, *Gast*, *Gkn1*, *Gkn2*, *Gkn3*, *Mki67*, *Lgr5*, *Lpo*, *Muc2*, *Muc6*, *Nox1*, *Pdia3*, *Pdia6*, *Pdx1*, *Qsox1*, *Sod1*, *Sod2*, *Sod3*, *Spdef*, *Tff1*, *Tff2*, *Tff3*, and *Zg16* expression in different parts of the murine intestine, i.e., proximal, medial, and distal parts of the duodenum (pD, mD, dD), middle section of the jejunum (J), distal ileum (I), and proximal/ascending colon (aC). Extracts of 10 female wild-type (WT, black bars) and 10 female *Tff1*^KO^ mice (white bars) were investigated. The number of amplification cycles is given after each gene. The relative gene expression levels were normalized against β-actin (*Actb*, 23x or 24x). Significances are indicated by asterisks (*, *p* ≤ 0.05; **, *p* ≤ 0.01; ***, *p* ≤ 0.001).

Relative gene expression levels were monitored in six different regions of the intestinal tract of *Tff1*^KO^ mice as well as their corresponding wild-type littermates (Figure 1).

The expression profiling (Figure 1) included transcripts encoding of TFF peptides (*Tff1*, *Tff2*, *Tff3*); gastrokines (*Gkn1*, *Gkn2*, *Gkn3*); goblet cell products (*Fcgbp*, *Clca1*, *Muc2*, *Zg16*); disulfide isomerases (*Agr2*, *Pdia3*, *Pdia6*, *Qsox1*); the mucin Muc6; glycosylation enzymes (*A4gnt*, *Fut2*); enzymes involved in the metabolism of ROS (*Nox1, Duox2*, *Lpo*, *Sod1*, *Sod2*, *Sod3*); transcription factors (*Cdx1*, *Cdx2*, *Pdx1*, *Spdef*); the hormone gastrin (*Gast*); the stem cell marker Lgr5, and the proliferation marker Ki67 (*Mki67*).

Generally, four kinds of gene expression profiles were observed within the intestine: (i) genes expressed in about equal amounts along the intestine (*Cdx1*, *Cdx2*, *Fcgbp*, *Clca1* (previously: *Gob5*), *Mki67*, *Lgr5*, *Muc2*, *Pdia3*, *Pdia6*, *Sod1*, *Sod2*, *Sod3*, *Tff3*, *Zg16*); (ii) genes with a maximum expression in the duodenum (*Gast*, *Gkn1*, *Gkn2*, *Gkn3*, *Pdx1*); (iii) genes, whose expression peaked in both the duodenum and the colon (*A4gnt*, *Agr2*, *Fut2*, *Muc6*, *Spdef*, *Tff1*, *Tff2*); (iv) genes with maximum expression in the colon (*Duox2*, *Lpo*, *Nox1*).

The most significant differences between wild-type and *Tff1*^KO^ mice were observed for the following genes (other than *Tff1*): *Gkn1*, *Gkn2*, *Clca1* (previously: *Gob5*), *Lgr5*, *Nox1*, *Spdef*, and *Tff2*. Here, differences were considered as being relevant when significance (*) was observed in at least two different regions or high significance (**) was observed in at least one region.

### 2.2. Expression Profiling of Tffs in the Murine Pancreas, Liver, and Lung (RT-PCR Analysis)

**Figure 2:**
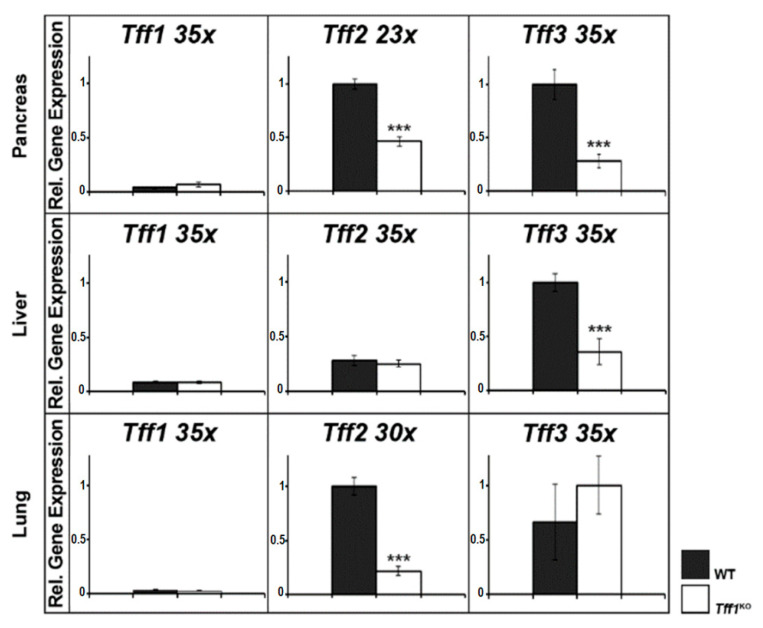
Semi-quantitative RT-PCR analyses (murine pancreas, liver, and lung). *Tff1*, *Tff2*, and *Tff3* expression was monitored in extracts of 10 female wild-type (WT, black bars) and 10 female *Tff1*^KO^ mice (white bars). The number of amplification cycles is given after each gene. The relative gene expression levels were normalized against β-actin (*Actb*; pancreas 27x, liver 24x, lung 21x). Significances are indicated by asterisks (***, *p* ≤ 0.001).

In the past, the expression of *Tff* genes was repeatedly documented to be changed in *Tff1*^KO^ mice as shown for the stomach [38,41] and the intestine (Figure 1). In order to complete these studies concerning other organs known for their *Tff* expression, we also investigated the pancreas, liver, and lung (Figure 2).

In the pancreas, both *Tff2* and *Tff3* expression were significantly reduced in *Tff1*^KO^ mice. In contrast, in the liver of *Tff1*^KO^ mice, only *Tff3* expression was significantly decreased, whereas in the lung of *Tff1*^KO^ mice, only *Tff2* expression was significantly down-regulated.

### 2.3. Protein Analysis of the Murine Duodenum

**Figure 3:**
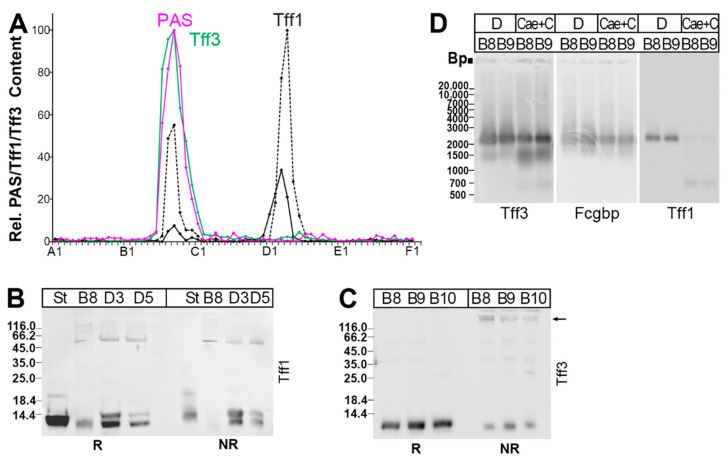
Analysis of a murine duodenal extract (complete duodena from four animals). The elution profile after SEC on a Superdex 75 HL column as well as the distribution of Tff2 have been reported previously [22]. (**A**) Distribution of the relative Tff1 (black) and Tff3 contents (green) as determined via Western blot analysis under reducing conditions and semi-quantitative analysis of monomeric band intensities. For Tff1, a regular band (black drawn line) and a somewhat shortened band (black dashed line) were analyzed separately. For comparison, the fractions were analyzed for their mucin content using the PAS reaction (pink); (**B**) 15% SDS-PAGE under reducing (R) and non-reducing (NR) conditions (post-in-gel reduction), respectively, and Western blot analysis of the high-molecular-mass fraction B8 and the low-molecular-mass fractions D3 and D5 concerning Tff1. As a control, fraction D1 from a murine stomach extract (St; [22]) was analyzed. (**C**) Analysis of the high-molecular-mass fractions B8–B10 concerning Tff3; (**D**) 1% AgGE and Western blot analysis of the high-molecular-mass fractions B8 and B9 concerning Tff3, Fcgbp, and Tff1, respectively (D, duodenal extract; Cae+C, extract from caecum plus total colon). Relative standard: DNA ladder (base pairs).

As *Tff1* transcripts were not detected in the small or large intestine in the past [42], we checked the positive RT-PCR results concerning *Tff1* (Figure 1) on the protein level. Here, a duodenal extract was separated via SEC, as reported previously [22], and analyzed for its Tff1 content (Figure 3). As a positive control, the Tff3 content was also determined.

Two Tff1 forms were detectable. In the high-molecular-mass region, it was mainly a slightly shortened Tff1 band that was found under reducing conditions (Figure 3A,B), which is barely detectable under non-reducing conditions, indicative of a disulfide-linked heterodimer (Figure 3B). In contrast, in the low-molecular-mass region, a regular and a shortened band were present (Figure 3A,B), which were both detectable also under non-reducing conditions, indicative of monomeric Tff1 (Figure 3B).

Tff3 appeared mainly in a high-molecular-mass form, and only minute amounts of a low-molecular-mass form were present (Figure 3A). From the high-molecular-mass form, Tff3 could be released after reduction (Figure 3C), and this band was partially shifted after non-reducing SDS-PAGE (arrow in Figure 3C).

As human TFF3 is known to form disulfide-linked TFF3-FCGBP heterodimers [10,15,45], we checked if Tff3-Fcgbp was detectable in the high-molecular-mass region (Figure 3D). Clearly, Tff3-Fcgbp was present in the duodenum. Furthermore, Tff1-Fcgbp was also detectable (Figure 3D).

**Figure 4:**
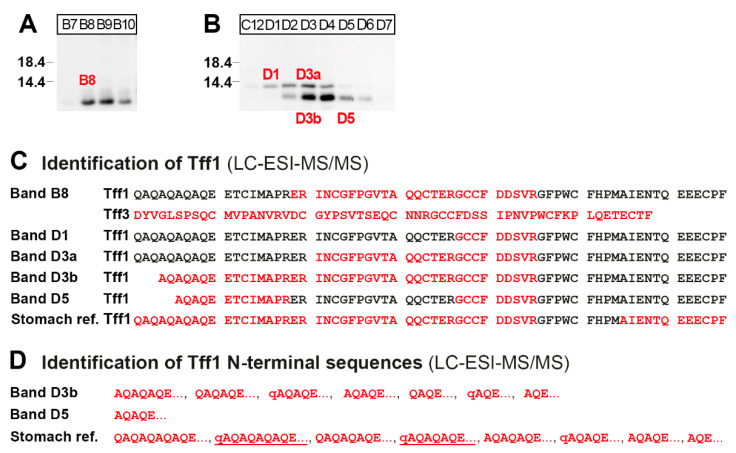
Proteome analysis of the high- and low-molecular-mass forms of Tff1 in a duodenal extract (fractions B8, and D1, D3, and D5 from Figure 3). (**A**,**B**) SDS-PAGE under reducing conditions of the high-molecular mass fractions B7–B10 (**A**) and the low-molecular-mass fractions C12–D7 (**B**) and Western blot analysis concerning Tff1. Fractions B8, D1, D3, and D5 were then separated via preparative reducing of SDS-PAGE, and after Coomassie staining, bands termed B8, D1, D3a, D3b, and D5 were excised (marked in red). (**C**) Results of the proteome analyses after tryptic in-gel digestion of bands B8, D1, D3a, D3b, and D5. Identified regions in Tff1 are shown in red. In B8, Tff3 was also identified. The results of the Tff1 reference (from a stomach extract) are also shown. The longest N-terminal sequences identified are shown. (**D**) Identification of heterogeneous Tff1 N-terminal sequences in bands D3b and the stomach reference (q indicates a pyro-Glu residue). The predominant sequences are underlined.

In order to verify the different Tff1 immunoreactive bands in the high- and low-molecular-mass regions (Figure 3A,B), the corresponding bands from fractions B8, D1, D3, and D5 (Figure 4A,B) were eluted and Tff1 was identified via bottom-up proteomics (Figure 4C). As a reference for the Tff1 sequence, Tff1 was isolated from the high-molecular-mass region of a murine stomach extract described previously [22] and analyzed in parallel (Figure 4C). For comparison, Tff3 was also identified in band B8 (Figure 4C).

### 2.4. Protein Analysis of the Murine Large Intestine

**Figure 5:**
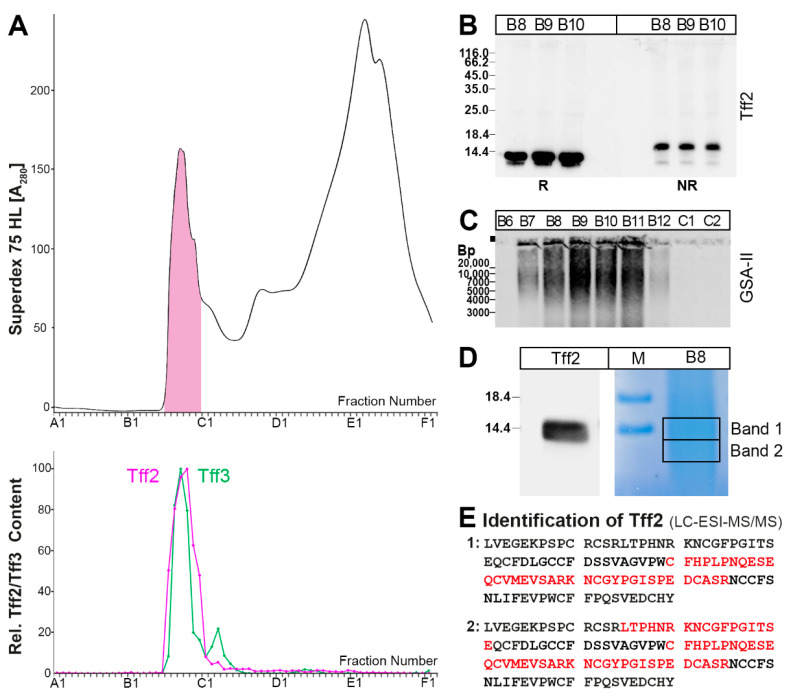
Analysis of a murine caecum plus total colon extract (single individual). (**A**) Elution profile after SEC on a Superdex 75 HL column as determined via absorbance at 280 nm (PAS-positive mucin fractions: pink). Underneath: distribution of the relative Tff2 (red) and Tff3 contents (green) as determined via Western blot analysis under reducing conditions and semi-quantitative analysis of the monomeric band intensities; (**B**) 15% SDS-PAGE under reducing (R) and non-reducing (NR) conditions (post-in-gel reduction), respectively, and Western blot analysis of the high-molecular-mass fractions B8–B10 concerning Tff2; (**C**) 1% AgGE and Western blot analysis of the fractions B6–C2 concerning Muc6 (lectin GSA-II). Relative standard: DNA ladder (base pairs). (**D**) SDS-PAGE under reducing conditions of fraction B8. Shown is a Western blot analysis concerning Tff2 and in parallel, Coomassie staining. Bands 1 and 2 were excised for proteome analysis. (**E**) Results of the proteome analysis after tryptic in-gel digestion of bands 1 and 2. Identified regions in Tff2 are shown in red.

In contrast to the positive RT-PCR analysis presented in Figure 1, *Tff2* mRNA was not observed in the murine large intestine in the past [42]. Thus, we checked Tff2 synthesis in the large intestine on protein level (Figure 5). As a positive control, the Tff3 content was analyzed.

Tff2 was exclusively detectable in a high-molecular-mass form (Figure 5A), which can be released under reducing as well as non-reducing conditions (Figure 5B). This high-molecular-mass region also contains Muc6, as detected via staining with the lectin GSA-II (Figure 5C). Furthermore, the presence of Tff2 was verified via bottom-up proteomics in the high-molecular-mass fraction B8 after reducing SDS-PAGE (Figure 5D,E).

Tff3 exists in a high-molecular-mass form (Figure 5A). After AgGE, this form was mainly identified as Tff3-Fcgbp (Figure 3D). However, there was also a second Tff3-positive band with a somewhat lower molecular mass, which was not positive with the anti-Fcgbp antiserum used (Figure 3D).

## 3. Discussion

### 3.1. Expression Profiling along the Murine Intestinal Tract: Indications for Different Mucosal Protection Systems

As controls, expression profiles of the genes encoding the transcription factors Cdx1, Cdx2, and Pdx1 were monitored (Figure 1). Expression of the intestine-specific genes *Cdx1* and *Cdx2* is rather uniform with a slight upregulation in the colon. In contrast, the transcription factor Pdx1 is known to regulate, in particular, endocrine differentiation in the gastric antrum, pancreas, and duodenum [46], which is in agreement with its expression in all three regions of the duodenum. Furthermore, expression of the hormone gastrin, particularly in the proximal duodenum, is as expected.

Generally, the expression profiling of genes encoding proteins known to play a role in the mucosal innate immune defense, such as Tff peptides, gastrokines, mucins, Fcgbp, and enzymes involved in the metabolism of ROS, point to different mucosal protection systems along the murine intestinal tract. On the one hand, secretory products of goblet cells protect the entire intestine (basic protection system). On the other hand, there are additional, specific protection systems, particularly in the proximal duodenum as well as the proximal colon.

#### 3.1.1. Basic Protection of the Entire Intestinal Tract by Goblet Cell Products (Muc2, Tff3-Fcgbp)

Genes encoding typical secretory proteins of goblet cells, i.e., *Clca1* (previously: *Gob5*), *Fcgbp*, *Muc2*, *Tff3*, and *Zg16*, show a rather uniform expression profile along the murine intestine (Figure 1). This result is not surprising as it reflects the common view that the complete intestine is protected by a mucus barrier produced by goblet cells, Muc2 being the predominant mucin. Another known component is the Tff3-Fcgbp heteromer, which has been demonstrated, e.g., in duodenal and colonic extracts (Figure 3D), and presumably plays a role for the mucosal innate immune defense [14,47].

For comparison, Tff3 was identified via proteomics in the high-molecular-mass Tff3-Fcgbp complex of the duodenum after reduction (Figure 4C). Because of its abundance, the complete Tff3 sequence could be determined, indicating for the first time, unambigously cleavage of the signal peptidase after Ala-23 of the precursor.

Notably, the expression of *Spdef* increases towards the colon with an additional peak in the proximal duodenum (Figure 1). This might be due to the increasing percentage of goblet cells relative to the total number of epithelial cells from the duodenum to the colon, as the transcription factor Spdef regulates terminal differentiation of goblet cells [48]. The peak in the proximal duodenum is caused by additional *Spdef* expression in Brunner glands [48].

#### 3.1.2. Specific Protection of the Proximal Duodenum and the Colon by the Tff2/Muc6 Complex

In the intestine, *Tff2*, *Muc6*, and *A4gnt* are predominantly expressed in the proximal duodenum (Figure 1). This is in agreement with their known synthesis in Brunner glands [19,22,42], which are located in the proximal duodenum only and are usually not found beyond the entrance of the pancreatic duct [18,49]. Thus, co-expression of *Tff2*, *Muc6*, and *A4gnt* in Brunner glands allows formation of a lectin-mediated, high-molecular mass Tff2/Muc6 complex, as already demonstrated on the protein level in murine duodenal extracts [22]. When compared with the murine stomach [41], the expression of *Tff2*, *Muc6*, and *A4gnt* in the intestine is much lower.

Of particular note, the expression of *Tff2*, *Muc6*, and *A4gnt* was also detectable in the proximal colon (Figure 1). In the past, there were contradictory reports concerning *Tff2* transcripts in the murine colon. They were either not detected [42] or described as being expressed in colonic epithelial cells [50], which might reflect differences between different mouse strains. However, immunohistochemistry of the proximal colon of rats localized Tff2 strongly in the lower parts of the crypts [51]. Thus, we tested an extract from caecum plus total colon for the presence of Tff2 (Figure 5). We could clearly identify a high-molecular-mass Tff2/Muc6 complex (Figure 5A), which was positive for GSA-II (Figure 5C), indicative of terminal GlcNAc residues in Muc6 due to A4gnt activity (Figure 5C). Tff2 was also identified via proteomics (Figure 5E). Notably, we could identify Tff2 not only in band 1, which is equivalent to the band strongly immunoreactive for Tff2 (Figure 5D), but Tff2 was also clearly identified in a band below with only weak immunoreactivity (designated as band 2: Figure 5D). This band can also be seen in Figure 5B and probably represents a shortened variant, maybe missing a few amino acid residues at the N- or C-terminal. However, the question of the cellular origin of this Tff2/Muc6 complex arises, as goblet cells are not known for the synthesis of Tff2.

In the past, the unusual GlcNAc-residue typical of Muc6 was recognized in gastric MNCs and AGCs as well as in Brunner glands, but also in the deep crypt cells of the rat colon [52,53]. These cells were first described by Altmann as “deep crypt secretory (DCS)” cells, particularly in the rat ascending and transverse colon, which originate from precursor cells and typically secrete mucus [54]. Later on, these cells were recognized again, due to their expression of Agr2 (previously termed Gob4) and typical staining with Alcian blue [16,55]. Notably, these Alcian blue positive DCS cells can be clearly distinguished from goblet cells, the latter being characterized by the synthesis of Tff3 [16]. Alcian blue is known to stain acidic mucins, such as Muc6 in gastric MNCs and AGCs, which are the characteristic Agr2-expressing cells in the murine stomach [56]. This implies that the disulfide isomerase Agr2, probably together with the disulfide isomerases Pdia3 and Pdia6 [57], plays a major role in the folding of Muc6, particularly in gastric MNCs and AGCs, as well as in duodenal Brunner glands and colonic DCS cells. This assumption is supported by the RT-PCR analysis concerning *Agr2*, which peaks in the proximal duodenum and proximal colon (Figure 1). It is also in line with a recent report describing Agr2 as a marker of colonic DCS cells, whose expansion is regulated by interleukin (IL)-13 originating from type 2 innate lymphoid cells (ILC2) [58]. However, Agr2 has been reported to occur in all intestinal secretory cell types [16], but reaches its highest level in the proximal colon [17]. Taken together, the synthesis of a Tff2/Muc6 complex in the DCS cells of the colon is comparable with the situation in the gastric antrum (review: [40]). Of particular note, Lgr5^+^ stem cells are located at the base of both the colonic crypts and antral glands [59]. Thus, it is tempting to speculate that the Tff2/Muc6 complex protects these basal stem cells in the colon from microbial colonization via a highly viscous mucous plug. This is in agreement with the view that DCS cells are important components of the colonic stem cell niche [60]. In contrast, the Lgr5^+^ stem cells in the small intestine are protected by secretory products of the neighboring Paneth cells, which are lacking in the colon [59].

#### 3.1.3. Specific Protection of the Duodenum by Tff1 and Gastrokines

*Tff1* and the gastrokine genes *Gkn1*, *Gkn2*, and *Gkn3* are also expressed selectively in the duodenum (Figure 1), but at much lower levels when compared with the stomach [41]. The intestinal expression of *Tff1* was somewhat surprising, as *Tff1* transcripts were neither detected in the small nor the large intestine in the past [42]. As the expression of *Tff1* and gastrokines is not confined to the proximal duodenum, expression in goblet cells might be possible. This view is supported by the observation that Tff1 exists in a high-molecular-mass form (Figure 3A), which has been identified as Tff1-Fcgbp heterodimer (Figure 3D), Fcgbp being typically secreted by goblet cells. Tff1-Fcgbp has already been described as occurring in the murine as well as the human stomach [41,61]. Thus far, it is not clear why Tff1-Fcgbp is mainly present in the duodenum and hardly detectable in the colon (Figure 3D); a possible reason may be different types of goblet cells, which differ in their Tff1 synthesis.

Proteomics clearly verified the existence of different Tff1 entities in the duodenum in both the high- and low-molecular-mass range (Figure 4). The high-molecular-mass form mainly exists in a shortened variant (Figure 3A,B and Figure 4A). The low-molecular-mass forms consist of a normal and shortened forms, the latter missing up to seven amino acid residues at least at the N-terminal (bands D3b and D5; Figure 4C,D) when compared with the longest Tff1 form from the murine stomach as a reference (Figure 4C,D). The heterogeneities at the N-terminal of Tff1 in the duodenum as well the stomach are remarkable (Figure 4D). In the stomach, the Tff1 precursor is preferentially cleaved by signal peptidase after Ala-21 or after Ala-23, liberating an unusual N-terminal repetitive sequence starting with a pyro-Glu residue (qAQAQAQAQE… and qAQAQAQE…, respectively, Figure 4D), due to cyclization of an N-terminal Gln residue with the help of glutaminyl cyclase. Currently, it is not clear how the multiple N-terminally-truncated Tff1 forms in the duodenum are generated (Figure 4D); it is possible that alternative cleavages by signal peptidase occur after various Gln or Ala residues and degradation by aminopeptidases. Notably, two forms were identified also starting with a pyro-Glu residue (qAQAQE…, qAQE…, Figure 4D), indicating cleavage by signal peptidase after Ala-25 and Ala-27, respectively. However, artificial cyclization in the electrospray ionization source cannot be excluded [62].

However, only *Gkn3* expression is significantly higher in the proximal duodenum when compared with the medial and distal parts, which is in line with its documented expression in Brunner glands [63]. As *Gkn3* is characteristically co-expressed with *Tff2* and *Muc6*, not only in Brunner glands but also in gastric MNCs and AGCs [30,63], it might be possible that Gkn3 supports the protective Tff2/Muc6 complex via a yet unknown mechanism.

#### 3.1.4. Specific Protection of the Proximal Duodenum and the Colon by Epithelial Fucosylation

As a hallmark, *Fut2* is predominantly expressed in the proximal duodenum and the proximal colon (Figure 1). Fut2 regulates fucosylation of intestinal epithelial cells. On the one hand, epithelial L-fucose is used as a dietary carbohydrate for many bacteria. On the other hand, fucosylation inhibits infection, e.g., from *Salmonella typhimurium* [64]. Of particular note, microbiota induce intestinal epithelial fucosylation by triggering *Fut2* expression [65]. For example, *Bacteroides* have been shown to induce epithelial fucosylation by direct interaction [66,67,68,69]. In addition, *Fut2* expression can also be mediated indirectly by interleukin (IL)-22 and lymphotoxin α originating from type 3 innate lymphoid cells (ILC3) [64]. Fucosylation can, e.g., change the signaling of receptors such as TLR4 in the murine colon, which is essential for recovery from mucosal injury in vivo [65]. Furthermore, goblet cells can be distinguished according to their fucosylation pattern, such as intercrypt goblet cells in the colon [70]. In contrast, fucosylation deficiency in mice leads to colitis and adenocarcinoma [71].

*Bacteroides* have been shown to accumulate in the murine proximal duodenum and also the colon [6]. Starting with the caecum, anaerobic genera appear in the murine lower alimentary tract and there is an increase in the richness (amount of different phylotypes) at this point [6]. These could be the reasons why *Fut2* expression peaks in the proximal duodenum and the proximal colon (Figure 1).

#### 3.1.5. Specific Protection of the Proximal Duodenum and Particularly the Colon by ROS-Generating Enzymes

Extracellular ROS such as the relatively stable (10^−2^–10^−3^ s) but weak oxidant H_2_O_2_ and the instable (10^−5^ s) superoxide are not only used by immune cells but also by mucous epithelia for innate immune defense against microorganisms [24,25,26]. H_2_O_2_ is diffusible and preferentially reacts with thiols from cysteine residues, whereas the superoxide anion cannot diffuse through membranes [25]. In the gastrointestinal tract, Duox2 typically generates extracellular H_2_O_2_ [25,26,72], which is used by Lpo to produce the highly microbicidal hypothiocyanite (DUOX/H_2_O_2_/LPO/SCN^−^ system) [28]. Of particular note, Duox2 and Lpo are differentially expressed in the colonic crypts of mice, i.e., *Duox2* expression is located at the upper crypt quintile, whereas *Lpo* transcripts are present in the basal quintile, where stem cells reside [73].

Expression of *Lpo* in the proximal colon (Figure 1) is an indication for a need for specific protection of these locations, controlling non-invasive pathogen colonization of the mucus, and might complement other protection systems such as fucosylation. The appearance of anaerobic genera and the increasing richness, particularly in the colon [6], would easily explain the large amounts of both Duox2 and Lpo needed in order to generate sufficient microbicidal SCNO^−^.

Furthermore, maximal expression of *Nox1* in the proximal colon (Figure 1) is in line with the drastically increasing number of bacteria in this region and fits with previous reports [25,26]. Notably, *Nox1* is most highly expressed in the lower two thirds of colon crypts [74] and thus might be specifically suited to protect the stem and precursor cells and/or enhance regeneration processes. However, the superoxide generated by Nox1 can be used to produce SCNO^−^ only in the presence of Sod3 and Lpo; the latter is also expressed at the basal quintile of the crypts [73].

Sod3 is typically present in luminal fluids as well as in the extracellular matrix and protects against oxidative stress-induced injury. Thus, it is not surprising that the entire intestine is protected by Sod3 (Figure 1). For comparison, the expression of *Sod1* and *Sod2* were also determined (Figure 1), encoding intracellular superoxide dismutases. These transcripts are far more abundant than that of *Sod3* and were detectable all along the intestine.

#### 3.1.6. Summary of the Different Mucosal Protection Systems along the Murine Intestine

**Figure 6:**
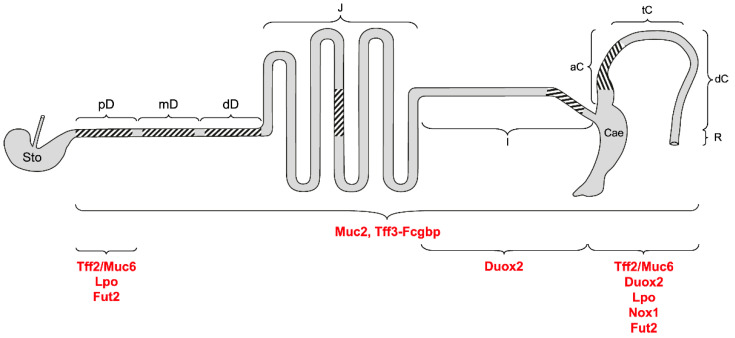
Schematic structure of the murine intestine and its different mucosal protection systems. Shown are stomach (Sto); proximal (pD), medial (mD), and distal parts of the duodenum (dD); jejunum (J); ileum (I); caecum (Cae); ascending/proximal (aC), transverse/medial (tC), and descending/distal colon (dC); rectum (R). The regions investigated in this study via RT-PCR are hatched. The predominant localization of the different intestinal protection systems is indicated.

In Figure 6, the different protection systems of the murine intestine are summarized. Clearly, besides a basic protection by goblet cell products along the entire intestinal tract, specific systems have evolved, particularly protecting the proximal duodenum (mainly Brunner glands secretions) and the proximal colon (particularly secretory products of DCS cells). The distal colon was not studied here.

### 3.2. Transcriptional Changes in the Intestine of Tff1^KO^ Mice

Major changes in *Tff1*^KO^ mice were the significantly increased expression of both *Gkn1* and *Gkn2* in the proximal duodenum (Figure 1). Based on the reported mitogenic activity of a Gkn1 fragment [75], an expected higher Gkn1 concentration might be responsible for the thickened villi of the small intestinal mucosa and the presence of inflammatory cells, as described previously [38]. However, macroscopically, we could observe intestinal abnormalities in the proximal duodenum only, i.e., a thickening. The significantly increased expression of *Gkn2* in *Tff1*^KO^ mice might result in secretion of a disulfide-linked Gkn2 homodimer as being detectable in the gastric antrum of *Tff1*^KO^ mice [41]. The biological function of such a Gkn2 form, particularly in *Tff1*^KO^ mice, is not known currently.

An additional hallmark is the significant down-regulation of *Tff2* in the proximal colon of *Tff1*^KO^ mice (Figure 1). Theoretically, this could diminish protection of the colonic stem cells by the Tff2/Muc6 complex and could lead to increased susceptibility of *Tff1*^KO^ mice to DSS-induced colitis, similarly to what has been reported for *Tff2*^KO^ mice [76]. However, *Tff1*^KO^ mice show the same response in a DSS colitis model as wild-type mice [50]. Thus, one might speculate that the significant up-regulation of *Nox1* expression, particularly in the proximal colon of *Tff1*^KO^ mice (Figure 1), could compensate for the reduced protection from bacterial colonization by the Tff2/Muc6 complex.

There is a tendency for up-regulated *Tff3* expression in the duodenum and the proximal colon of *Tff1*^KO^ mice (Figure 1), which is in agreement with a previous report [38]. Furthermore, also gastrin expression shows a tendency for up-regulation in the duodenum of *Tff1*^KO^ mice (Figure 1). This is in contrast to the antrum, where *Tff1*^KO^ mice show statistically significant down-regulation of *Gast* [41].

Notably, there is a generally up-regulated expression of *Clca1* (previously *Gob5*) and *Lgr5* in the entire intestine of *Tff1*^KO^ mice, which is significant for both genes in the ileum and colon, and additionally for *Lgr5* in the proximal duodenum (Figure 1). The up-regulation of the stem cell marker, *Lgr5*, would be in agreement with reports that TFF1 is able to control cell differentiation by regulating the balance between cell proliferation and death (anti-proliferative and anti-apoptotic effects of TFF1) [38,77,78,79]. Furthermore, *Agr2* and *Sod1* also show a tendency toward up-regulation in *Tff1*^KO^ mice (Figure 1). Up-regulation of *Agr2* might be a response to latent endoplasmic reticulum (ER) stress [80,81], which could be due to the unfolded protein response (UPR) activated in *Tff1*^KO^ mice [82].

### 3.3. Tff Expression Is Cross-Regulated in the Pancreas, Liver, and Lung of Tff1^KO^ Mice

In the past, coordinate regulation of *Tff* genes was observed (review [83]), which is due to the clustered organization of the three *Tff* genes in a head-to-tail orientation within a 40 kb region on chromosome 17q [84]. For example, *Tff2* expression is significantly down-regulated in the stomach of *Tff1*^KO^ mice, particularly in the corpus [38,41,85]. Generally, the linear organization of the *Tff* genes reflects their spatial distribution along the GI tract, strongly suggesting the existence of a locus control region [84]. This region may be affected in the *Tff1*^KO^ mice, resulting in down-regulation of *Tff2* and *Tff3*, particularly in organs of the GI tract. However, epigenetic mechanisms play a major role in the regulation of *Tff* expression [84].

Here, we show that *Tff2* is also significantly down-regulated in the pancreas and the lung of *Tff1*^KO^ animals (Figure 2). In both of these organs, Tff2 is the predominant TFF peptide. The down-regulation of pancreatic *Tff2* expression is in agreement with a previous report [38]. Loss of pancreatic *Tff2* has been shown to promote formation of intraductal papillary mucinous neoplasms in mice [86]. Thus, it might be possible that *Tff1*^KO^ mice exhibit a similar phenotype due to a secondary effect. Furthermore, as *Tff2*^KO^ mice were reported to have compromised lung structure and function [87], it would also be interesting to investigate the lung of *Tff1*^KO^ animals in detail.

In the liver of *Tff1*^KO^ mice, *Tff3* expression is significantly down-regulated (Figure 2). *Tff3* is moderately expressed in biliary epithelial cells [88] and dramatic down-regulation of *Tff3* expression was observed in a murine model of type II diabetes [89]. *Tff3* down-regulation is also correlated with a fatty-liver phenotype [90]. Furthermore, *Tff3*^KO^ mice show altered liver lipid metabolism [91]. Thus, it would be interesting to check if a similar phenotype occurs in *Tff1*^KO^ mice.

## 4. Materials and Methods

### 4.1. Animals

Animal care and experimental procedures were conducted in compliance with the Directive 2010/63/EU of the European parliament and of the council of 22 September 2010 on the protection of animals used for scientific purposes, the German Animal Welfare Act, and the regulations on the welfare of animals used for experiments or for other scientific purposes in their currently valid versions. In the course of these studies, *Tff1*^KO^ mice and their corresponding wild-type littermates (mixed 129/Sv and C57BL/6 background) described previously [41,92] were investigated at the age of six weeks (Landesverwaltungsamt Sachsen-Anhalt; license number: 203.m-42502-2-1722 UniMD; 18 May 2022). Mice heterozygous for *Tff1* were originally obtained from Dr. M.-C. Rio and Dr. C. Tomasetto (IGBMC, Illkirch, France) [38]. Furthermore, for protein analysis, adult wild-type animals with a mixed 129/Sv and C57BL/6 background at the age of 16 weeks were used as described previously described [6,93] (Animal Welfare Officer of the Medical Faculty of the Otto-von-Guericke University Magdeburg; license number: IMMC-TWZ-01; 1 January 2015).

### 4.2. RNA Extraction, PCR Analysis

Isolation of total intestinal, hepatic and pulmonary RNA, respectively, (TRIzol^TM^ Reagent; ambion by life technologies, Carlsbad, CA, USA), of pancreatic RNA (RNA Mini Kit, Bioline, Heidelberg, Germany), as well as RT-PCR (reverse transcriptase: Takara Bio Europe, Saint Germain en Laye, France) were as previously described in detail [93,94,95].

The specific primer pairs used for RT-PCR have been published previously (*A4gnt,* MB2430/MB2431; *Actb*, MB2658/MB2659; *Fcgbp*, MB2448/MB2449; *Gast*, MB2450/MB2451; *Gkn1*, MB2450/MB2451; *Gkn2*, MB2456/MB2457; *Gkn3*, MB2656/MB2657; *Muc6*, MB2320/MB2321; *Pdia3*, MB2744/MB2745; *Pdx1*, MB2464/MB2474; *Tff1*, MD7/MD8; *Tff2*, MB2306/MB2307; *Tff3*, MB2470/MB2471) [22,41,93,95] or are listed in Table 1. All primer pairs used are intron-spanning.

Semi-quantitative evaluation of the relative expression levels of the selected genes was performed using the GeneTools analysis software (Version 4.3.17.0, Syngene Bioimaging, Cambridge, UK), as previously described in detail [93]. Generally, the relative intensities were normalized against the relative intensities of the *Actb* transcripts (intestine 23 or 24, liver 24, lung 21, and pancreas 27 amplification cycles, respectively) and the highest value (mean) within each series was set to 1. If no robust signal was obtained (Figure 2: *Tff1*, *Tff2*/liver), an external signal was used as standard and set to 1. The statistical analysis using Student’s t-test was performed with the Excel 2019 software package (Microsoft, Syracuse, NY, USA). Error bars represent ±SEM. Significant differences between the mean values between wild-type and *Tff1*^KO^ mice are indicated by asterisks (*p* ≤ 0.05: significant, *; *p* ≤ 0.01: highly significant, **; *p* ≤ 0.001: extremely highly significant, ***).

### 4.3. Extraction of Proteins, Protein Purification via SEC

Extraction and fractionation via SEC of total duodena from 4 animals were previously described in detail [22]. Furthermore, the caecum plus total colon from a single individual was collected and extracted with a 6.2-fold amount (*w*/*v*) of buffer (30 mM NaCl, 20 mM Tris-HCl pH 7.0 plus protease inhibitors) in a Precellys^®^ 24 lyser/homogenizer, similarly to previous descriptions (aqueous extracts) [10,96]. A total of 5 mL of the extracts were fractionated via SEC with the ÄKTA^TM^ FPLC system (Amersham Biosciences, Freiburg, Germany) as described (fraction numbering: A1–A12, B1–B12, etc.), using a HiLoad 16/600 Superdex 75-prep-grade column (S75HL; 20 mM Tris-HCl pH 7.0, 30 mM NaCl plus protease inhibitors; flow rate: 1.0 mL/min; 2.0 mL fractions) [97].

### 4.4. SDS-PAGE, AgGE, and Western Blot Analysis

Denaturing SDS-PAGE under reducing and non-reducing conditions, respectively, native AgGE, and Western blot analysis were described previously [10,41,98,99]. When indicated, gels after non-reducing SDS-PAGE were subjected to post-in-gel reduction with 1% mercaptoethanol at 50 °C for 2 min, according to a previous report [97]. As a relative standard for non-denaturing AgGE, a DNA ladder was used as specified previously [15].

Murine Tff1 and Tff2 were detected with the affinity-purified polyclonal antisera anti-mTff1-1 [94] and anti-TFF2 (PA5-75670; Invitrogen by Thermo Fisher Scientific Baltics UAB, Vilnius, Lithuania), respectively. For the detection of Tff3, the polyclonal antiserum, anti-rTff3-1 [100], was used. Fcgbp was detected with a polyclonal antiserum against a fragment of rat Fcgbp kindly provided by Prof. Jürgen Seitz (Philipps University, Marburg, Germany) [101] and the mucin Muc6 with the biotinylated lectin GSA-II from *G. simplicifolia*, as reported [97,102].

### 4.5. Identification of Proteins via Bottom-Up Proteomics

For protein identification, gel bands were excised and subjected to tryptic digestion, followed by liquid chromatography coupled to electrospray ionization and tandem mass spectrometry (LC-ESI-MS/MS). The data obtained were processed and analyzed with a search engine, as described in detail previously [15]. For N-terminal glutamine residues, cyclization to pyroglutamic acid (pyro-Glu) was also taken into account. This is a posttranslational modification, which is typical of some TFF peptides, but cyclization of free Gln and Glu can also occur in the electrospray ionization source [62].

## 5. Conclusions

In this study, different mucosal protection systems were systematically localized along the murine intestine (Figure 6). Remarkably, the evolutionary old Muc6/A4gnt/Tff2 system is not restricted to the stomach and Brunner glands, but also protects the deep crypts, particularly of the proximal colon. In the latter, the expression of *Nox1* and of *Lpo* also culminate. A systematic investigation of the distinct parts of the colon is a future challenge. This might help to increase the understanding of the differences in the carcinogenesis in the distinct colonic regions. Furthermore, we characterized Tff1-Fcgbp heterodimers, which are probably involved specifically in duodenal innate immune defense. Notably, *Tff1*-deficient animals show significantly up-regulated *Gkn1* and *Gkn2* expression in the proximal duodenum when compared with the wild-type. Furthermore, the expression of *Tff* genes is cross-regulated, particularly in the GI tract, leading to a down-regulation of *Tff2* and *Tff3* in *Tff1*^KO^ animals.

## Figures and Tables

**Table 1:** Oligonucleotides used for RT-PCR analysis and calculated size of the products.

Genes Accession No.	Primer No.	Primer Pairs	Nucleotide Positions	Annealing T Size (bp)
*Agr2* NM_011783.2	MB2190 MB2191	GTCTGCAATCCTGCTTCTTGT GTCTTTAGCAGCTTGAGAGCTT	70–90 570–549	60 °C 501
*Cdx1* NM_009880.4	MB2354 MB2355	GGACGCCCTACGAATGGAT ACCAGATCTTTACCTGCCGC	489–507 704–685	60 °C 216
*Cdx2* NM_007673.3	MB2352 MB2353	AGCCAAGTGAAAACCAGGACA GATGCTGTTCGTGGGTAGGA	799–819 1320–1301	60 °C 522
*Clca1* NM_017474.2	MB2176 MB2177	CTTATCACCTGGACAACGCA TGGTCCCTGAGATCAACGAT	1589–1608 2436–2417	60 °C 848
*Duox2* NM_001362755.1	MB2726 MB2727	GCCTGTCGAGTCTCGTTCAT CCGCAAGAAGGTGATGAGGT	2934–2953 3383–3364	60 °C 450
*Fut2* NM_001271993.1	MB2468 MB2469	CTCCCCCGGGATCCTTATCT GTGGTAATTCTGCCACGGG	252–271 699–681	60 °C 448
*Lgr5* NM_010195.2	MB2492 MB2493	GTCTCCTACATCGCCTCTGC AGAAGGGTTGCCTACGAACG	538–557 1133–1114	60 °C 596
*Lpo* NM_080420.3	MB3005 MB3006	GGCTGCCACGGGAGGTCAA TTATAGGGTGGTGTGGGGCA	11–29 906–887	60 °C 896
*Mki67* NM_001081117.2	MB2458 MB2459	AGAGCTAACTTGCGCTGACTG TCTTGAGGCTCGCCTTGATG	129–149 618–599	60 °C 490
*Muc2* NM_023566.4	MB2178 MB2179	GGCTCTACAGACAAGCAGAC CATGAAGGTATGGTCAGGGC	1329–1348 2141–2122	60 °C 813
*Nox1* NM_172203.2	MB2883 MB2884	AAGTTTCTCTCCCGAAGGACC CCCTCAAGAAGGACAGCAGA	74–94 387–368	60 °C 314
*Pdia6* NM_027959.4	MB2991 MB2992	TGGTCGGACGAGATCTGACA TGAGACGCTGAGGTTCACTG	806–825 1513–1494	60 °C 708
*Qsox1* NM_001024945.1	MB2546 MB2547	TATAGTGAGGCCCACCCACA GTACATCTAGGGCAGTGGCTC	1370–1389 1895–1875	60 °C 526
*Sod1* NM_011434.2	MB2837 MB2838	CGGTGAACCAGTTGTGTTGTC GGTCTCCAACATGCCTCTCT	174–194 349–330	60 °C 176
*Sod2* NM_013671.3	MB2839 MB2840	CTGGACAAACCTGAGCCCTA GTTGTTCCTTGCAATGGGTCC	510–529 728–708	60 °C 219
*Sod3* NM_011435.3	MB2184 MB2185	CTGCTGCTCGCTCACATAA CGCCTGGAGACATCTATGC	119–137 1077–1059	60 °C 959
*Spdef* NM_013891.4	MB2200 MB2201	AAGATATTGAGACGGCCTGC TGTCTATCTGGGACCTTGGG	790–809 1528–1509	60 °C 739
*Zg16* NM_026918.3	MB2995 MB2996	CCTCGGCCTCTGCTAATTCC CCTGGATCACAGATTCCCCG	85–104 339–320	60 °C 255

## Abbreviations

AGC	Antral gland cell
AgGE	Agarose gel electrophoresis
FCGBP	IgG Fc binding protein
GI	Gastrointestinal
GKN	Gastrokine
MNC	Mucous neck cell
SEC	Size exclusion chromatography
SDS-PAGE	Sodium dodecyl sulfate-polyacrylamide gel electrophoresis
TFF	Trefoil factor family
